# The Diagnostic Yield and Implications of Targeted Founder Pathogenic Variant Testing in an Israeli Cohort

**DOI:** 10.3390/cancers16010094

**Published:** 2023-12-24

**Authors:** Aasem Abu Shtaya, Inbal Kedar, Samar Mattar, Ahmad Mahamid, Lina Basel-Salmon, Sarit Farage Barhom, Sofia Naftaly Nathan, Nurit Magal, Noy Azulay, Michal Levy Zalcberg, Rakefet Chen-Shtoyerman, Ori Segol, Mor Seri, Gili Reznick Levi, Shiri Shkedi-Rafid, Chana Vinkler, Iris Netzer, Ofir Hagari Bechar, Liat Chamma, Sari Liberman, Yael Goldberg

**Affiliations:** 1 Recanati Genetics Institute, Rabin Medical Center—Beilinson Hospital, Petach Tikva 4941492, Israel; inbalkd@clalit.org.il (I.K.); linab@clalit.org.il (L.B.-S.); nmagal@clalit.org.il (N.M.); morse@clalit.org.il (M.S.); ofirhi1@clalit.org.il (O.H.B.); yaelgo43@clalit.org.il (Y.G.); 2 Unit of Gastroenterology, Lady Davis Carmel Medical Center, Haifa 3436212, Israel; ori_segol@clalit.org.il; 3 Department of Surgery B, Carmel Medical Center, Haifa 3436212, Israel; samarma@clalit.org.il (S.M.); ahmadma1@clalit.org.il (A.M.); 4 Faculty of Medicine, Tel Aviv University, Tel Aviv 6997801, Israel; 5 Felsenstein Medical Research Center, Petach Tikva 4920235, Israel; 6 Pediatric Genetics Unit, Schneider Children’s Medical Center of Israel, Petch Tikva 49202, Israel; 7 Genetics Institute, Soroka Medical Center, Beer Sheva 84101, Israel; michallevy@clalit.org.il; 8 Adelson School of Medicine, Department of Molecular Biology, Ariel University, Ariel 40700, Israel; rakefet.rel@gmail.com; 9 Kaplan Medical Center, Genetics Institute, Oncogenetic Clinic, Rehovot 7610001, Israel; 10 Genetics Institute, Rambam Health Care Campus, Haifa 31096, Israel; g_reznick@rmc.gov.il; 11 Department of Genetics and Metabolic Diseases, Hadassah Hebrew University Medical Center, Jerusalem 91120, Israel; shirish@hadassah.org.il; 12 Institute for Medical Genetics, Wolfson Medical Center, Holon 5822012, Israel; vinkler@wolfson.health.gov.il; 13 Oncogenetics Unit, Institute of Human Genetics, Chaim Sheba Medical Center, Tel Hashomer 52621, Israel; iris.netzer@sheba.health.gov.il; 14 Medical Genetics Institute, Shaare Zedek Medical Center, Jerusalem 9112102, Israel

**Keywords:** *BRCA1*, *BRCA2*, Lynch, *APC*, cancer, founder, Israel, yield

## Abstract

**Simple Summary:**

This study examined the efficacy and diagnostic yield of founder variant testing as an initial screening method for individuals with a personal or family history of cancer. The results indicate that this approach is ineffective and has a limited ability to detect significant germline variants. Given these findings, we raise doubts about the benefits of employing such testing and suggest a transition from a two-step approach to expansive genetic testing.

**Abstract:**

Founder pathogenic variants (PVs) are prevalent in Israel. This study investigated the current practice of offering cancer patients two-step genetic testing, starting with targeted testing for recurring founder PVs, followed, if negative, by next-generation sequencing. A total of 2128 subjects with cancer or a positive family history underwent oncogenetic testing with a panel of 51 recurring PVs at a tertiary medical center in March 2020–January 2023. Those with a known familial PV (n = 370) were excluded from the analysis. Among the remainder, 128/1758 (7%) were heterozygous for at least one variant, and 44 (34%) carried a PV of medium-high penetrance (MHPV). Cancer was diagnosed in 1519/1758 patients (86%). The diagnostic yield of founder MHPV testing was 2% in cancer patients and 4% in healthy individuals with a positive family history. It was higher in Ashkenazi Jews than non-Ashkenazi Jews and Arabs, but not over 10% for any type of cancer, and it was significantly higher in younger (<40 years) than older (>50 years) individuals (7% vs. 1%). Eighty-four of the heterozygotes (66%), mostly Ashkenazi Jews, harbored a low-penetrance variant (LPV) not associated with the diagnosed cancer, usually *APC* c.3902T>A. These findings question the advantage of two-step testing. LPVs should not be included in targeted testing because this can lead to an overestimation of the yield, and their detection does not preclude further comprehensive testing.

## 1. Introduction

An estimated 5% to 10% of all cancers can be attributed to inherited genetic conditions [1]. Oncogenetic testing is rapidly emerging as a pivotal tool for evaluating an individual’s inherited risk of developing cancer and for guiding personalized medical decisions. It is particularly beneficial for healthy individuals with a strong family history of specific cancers [2]. Genetic testing currently ranges from testing a single variant to whole genome sequencing.

A founder variant is a genetic alteration inherited from a common ancestor that is observed at a high frequency in a defined population. Founder variant testing (FVT) is especially relevant in populations that have a history of endogamy, are geographically isolated, or experienced historical events that contributed to the prevalence of specific variants. Founder PVs have been identified in several genes, including *BRCA1* and *BRCA2* in the Ashkenazi Jewish population, *MUTYH* in the European population and North African Jews [3], *HEXA* in Ashkenazi Jews and certain French-Canadian populations [4,5,6], and *HFE*, associated with hereditary hemochromatosis, in individuals of Northern European descent [7]. FVT streamlines the diagnostic process and is amenable to widespread use: it is relatively inexpensive, quick, and straightforward; the genotype-phenotype association has been studied; and it makes it possible for practitioners to reach a precise diagnosis without encountering variants of unknown significance (VUS) and without the need for time-consuming bioinformatics analysis. 

FVT is part of a two-step algorithm that was introduced as a more efficient approach to improve the diagnostic process and lower costs. It consists of targeted testing of recurring variants in a specific population, followed by more elaborate and expanded genetic testing. For patients with a pretest probability of 10% or more, if the first step is positive, no further testing is required. If the first step is negative, it is followed by next-generation sequencing (NGS)-based multigene panels, depending on the cancer type. For example, for breast, ovarian, and pancreatic cancer, the following panel is performed: *ATM, BRCA1, BRCA2, BRIP1, CDH1, CHEK2, MSH2, MLH1, MSH6, PMS2, EPCAM, NBN, NF1, PALB2, PTEN, RAD51C, RAD51D, STK11,* and *TP53*.

Before 2020, FVT was common practice in Israel, focusing mainly on 14 common founder PVs in *BRCA1/2* genes in Jews from various ethnicities. Thereafter, the Israel Ministry of Health modified the two-step genetic testing program to include 51 recurring pathogenic variants in 8 additional genes. The modified test includes pathogenic variants in *APC, CHEK2, MLH1, MSH2, MSH6, MUTYH, PMS2,* and *TP53*, which have been documented as prevalent in the Jewish and non-Jewish Israeli populations. Variants were selected based on previous publications or based on data collected in local genetic institutes (Table 1) [8,9,10,11]. In this manner, one test can be used for all ethnic groups in Israel. The word Jewish in Table 1 refers to Israeli Jews of different ethnicities other than AJ i.e., Kurdish, North African, Balkan, Bukharan, Iraqi, Greek, Yemenite, Ethiopian, and others. Among the variants included are the *APC* c.3920T>A with a carrier frequency of ~6.5% among AJ; the three *BRCA1* variants c.5266dupC, c.68_69delAG and c.2934T>G with carrier frequency of 0.45%, 0.75% and 1%, respectively; the *BRCA2* c.7579delG detected among ~1% of Ethiopian Jews, the *BRCA1* c.2934T>G among Iranian Jews. The *CHEK2* variants c.1283C>T are common among AJ (2.2%) and the c.499G>A common among Christian Arabs. Lynch syndrome-associated genes are also included, some of the most common variants included are the c.1906G>Ain *MSH2* and c.3959_3962delCAAG in *MSH6* (carrier frequency of 0.2% and 0.06%, respectively) among AJ. The *MUTYH* c.1187G>A and c.536A>G are very common (6%) among Moroccan Jews while the *TP53* c.541C>T is a recurrent PV in Muslim Arabs. 

Patients eligible for FVT must meet one of three criteria: 1. They received a diagnosis of breast, ovarian, metastatic prostatic, pancreatic cancer, or a Lynch syndrome-associated cancer; 2. They are otherwise healthy but have a significant family history of the same cancers, defined as the probability of variant detection of >10%, as calculated by the various risk stratification models. 3. They carry a known familial PV. 

Apart from PVs of medium-high penetrance (MHPVs) in *BRCA1/2,* testing also includes low-penetrance alleles, which have been frequently reported in patients with cancer, for example, *APC* c.3920T>A and *CHEK2* c.1283C>T [12] (Table 1).

The aim of the present study was to assess the still-unclear yield of FVT, the first of the two-step oncogenetic testing programs in Israel, and the spectrum of disease-causing recurring variants in an Israeli cohort of patients with cancer and healthy individuals with a significant family history of cancer or a known familial PV attending the Genetics Institute of Rabin Medical Center. Previous studies have evaluated the second step (expended NGS-based genetic testing) in the Israeli population in a cohort of breast and ovarian cancer patients, most of them were limited to specific ethnicities and found the diagnostic yield to be between 6-7% in ovarian and breast cancer patients [13] (PMID, Cohorts from other countries show similar results [14].

## 2. Patients and Methods

### 2.1. Study Population

This study included subjects referred to a tertiary university medical center for oncogenetic testing between March 2020 and January 2023. Patients had either a personal or family history of breast, ovarian, prostatic, or pancreatic cancer, gastrointestinal cancer, or Lynch syndrome-associated cancer. A significant family history was defined as a variant detection probability of more than 10% calculated by a risk stratification model such as BRCApro, CanRisk, PENN II, and PREMM5, depending on the cancer type [15,16,17,18,19]. Healthy individuals with a first-, second-, or third-degree relative with a known PV previous to testing were part of the original cohort, but they were excluded from the analysis of test yield.

Detailed data on demographic features, medical history, and personal and family history of cancer were collected in the course of genetic counseling. Ethnicity was self-reported and defined as the place of birth of the paternal and maternal grandparents. The study was approved by the institutional review board—Helsinki committee.

### 2.2. Genotyping

For step 1, FVT testing DNA was extracted by an automatic system (MagNa Pure LC for nucleic acid purification, Roche Ltd., Basel, Switzerland). Fifty-one germline genetic variants that have been implicated in an increased risk for breast, ovarian, prostatic, pancreatic, and colon cancer in the Israeli population (see list of variants in Table 1) were tested with the NanoChip^®^ ONCO PANEL 51 kit, NanoChip^®^ 400, and Nanogen technology (Savyon Diagnostics Ltd., Ashdod, Israel). 

The ONCO51 NanoChip^®^ assay is used to discriminate among 51 oncogenic variants. It consists of a cartridge comprised of an electrical microarray covered with a permeation layer embedded with streptavidin. The streptavidin serves to bind 5′ or 3′ biotinylated capture oligos to the permeation layer via an electrical attraction force. For each sample, amplicons created by dual multiplex polymerase chain reactions (PCRs) are applied to the array at specific pads, hybridizing with the captured oligos. Fluorescence reporting oligos hybridize to the “captured amplicons” in a sequential manner, following an imaging stage and thermal stripping. The fluorescence signals are quantitated, analyzed by pre-calibrated green-to-red signal ratio ranges, and processed to assign WT/HET/HOM genotypes for each variant. Each stripping action leaves the captured amplicons ready for the next reporting reaction. At the end of the reporting cycles, reverse bias washing is performed to remove any amplicon contamination, preparing the array for future use (NanoChip^®^ OncoPanel 51 Application Note, 2021).

## 3. Results

A total of 2128 subjects underwent genotyping during the study period (Table 2). Those who were tested because of a previously reported familial PV (cascade testing, n = 370) were excluded from the analysis. Among the remainder, 42% were Ashkenazi Jews, 41% were non-Ashkenazi Jews, 10% were of mixed descent (Ashkenazi Jew and other ethnicities), and 7% were Israeli Arabs (including Muslims, Christians, Druze and Bedouin). The male-to-female ratio was 1:5 across the entire cohort and among the different Jewish ethnic groups, and 1:10 in the Arab group. Around 16% of the cohort was below the age of 40 years, 18% were aged 40 to 50 years, and 66% were above age 50 years.

The cohort consisted of 1519/1758 patients (86%) with a personal history of cancer, and 239/1758 healthy individuals (14%) with a significant family history of cancer. Age at cancer diagnosis ranged from 22 to 94 years (mean 62±14 years). The most common malignancy was breast cancer in 904 patients (60%), followed by prostate cancer in 100 patients (7%), colorectal cancer in 88 patients (6%), ovarian cancer in 72 patients (5%), and pancreatic cancer in 64 patients (4%); the remaining 291 patients (19%) had other types, primarily tumors associated with Lynch syndrome. More than one type of cancer was found in 76 patients (5%). The most common combinations were breast and either colorectal or endometrial cancer (11 cases each). Among the individuals with a family history of cancer, the most common malignancy reported in the family was breast cancer (180/239, 75%).

In both Ashkenazi Jewish and non-Ashkenazi Jewish subjects, the number of cases of cancer increased with age. This was partly explained by the old age at cancer diagnosis of most patients. The low percentage of Israeli Arabs in the cohort suggested that the rate of testing in this group may have been low. 

**Figure 1:**
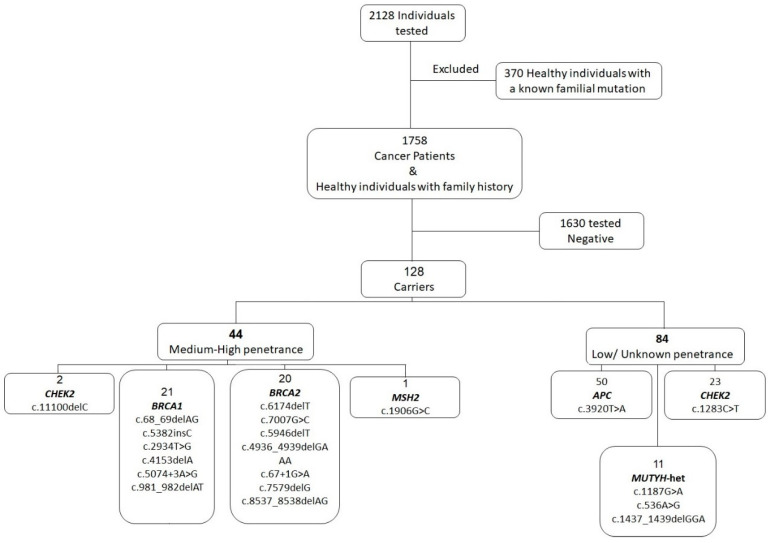
Results of targeted FVT in the cohort.

Targeted FVT, including high-, medium-, and low-penetrance variants, was positive in 128/1758 patients (7%) (Table 3a). The carrier detection rate dropped to 44/1758 (3%) when low-penetrance PVs (LPVs) of unclear clinical significance were excluded, mainly *APC*:c.3920T>A, *CHEK2*:c.1283C>T, and heterozygous *MUTYH* PV (Figure 1 and Table 3b).

MHPVs were detected in 44/128 carriers (34%) who were heterozygous for at least one variant and 34/1519 patients (2%) with cancer (Table 3b). There was no significant difference in the MHPV detection rate (below 5%) among patients with different types of cancer or among patients of different ethnicities. MHPVs were found mostly in patients with pancreatic cancer (5%), followed by patients with ovarian and prostatic cancers (4% each). By ethnicity, the MHPV yield was highest in Ashkenazi Jews: 10% in those with pancreatic cancer and 7% in those with ovarian cancer.

Three of the 96 Israeli Arab patients with cancer were heterozygotes (4%); two of them carried the MHPV c.5074+3A>G in *BRCA1,* previously reported as a common variant in the Arab population [20]. 

The rate of variant detection was about sevenfold higher in patients diagnosed at an earlier age. MHPVs were detected in 23% of subjects (cancer patients and healthy individuals) who were younger than 40, compared with 2% of individuals older than 50. 

The majority of genotyped heterozygotes (84/128, 66%) harbored LPVs. The most common LPV (50/84 patients) was *APC*:c.3902T>A; all carriers were Ashkenazi Jewish or partly Ashkenazi Jewish, except for one Israeli Arab with polyposis. The rate of detection in the Ashkenazi Jewish and the partly Ashkenazi Jewish groups was 5%. None of the heterozygotes had colorectal cancer, although 11 of the 50 carriers (22%) reported a family history of colorectal cancer.

The variant detection rate among patients diagnosed with any two cancers was 13%, decreasing to 1% when LPVs were excluded. Most of the LPVs detected did not match the tumor diagnosed (e.g., *APC* variants in individuals with breast and ovarian cancer). None of the patients with two cancers carried an MHPV matching either of the tumors/cancer syndromes (see Table 3a and Table 3b).

Among healthy individuals with significant family history and no known familial PV, 4% harbored an MHPV. All were Ashkenazi Jewish or partly Ashkenazi Jewish. None of the non-Ashkenazi Jews or Israeli Arabs who were tested because of a family history were carriers of any MHPV. 

**Figure 2:**
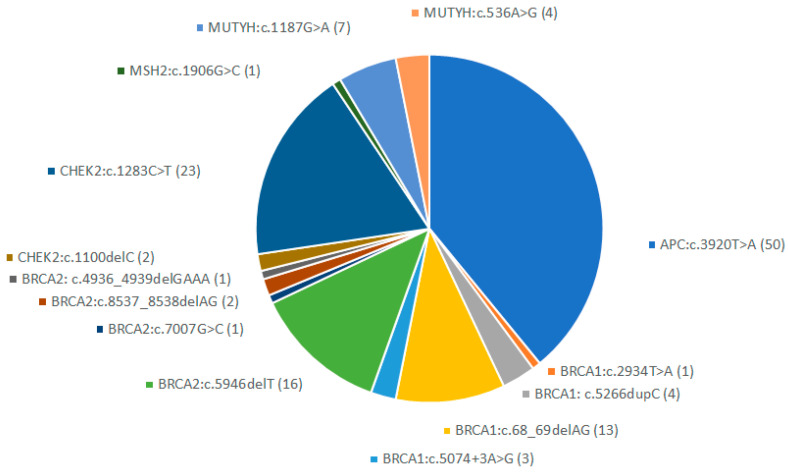
Founder variants detected in the cohort.

Fourteen of the 51 founder variants included in the panel appeared at least once. The remaining 37 were not detected in any patient or healthy individual in the entire cohort (Figure 2). The most common variant, *APC*:c.3902T>A, was detected 50 times, and *CHEK2*:c.1283C>T was detected 23 times. The most common *BRCA1* variant, detected 13 times, was c.68_69delAG, and the most common *BRCA2* variant, detected 16 times, was c.5946delT. No founder variants were detected in *MLH1, MSH6, PMS2,* or *TP53*.

## 4. Discussion

Israel’s diverse demographic makeup reflects a rich tapestry of cultures and backgrounds. As of the end of 2021, Israel’s population consisted of approximately 73.9% Jews, or around 6.982 million people, and approximately 21% Arabs, primarily Muslims with smaller Christian and Druze communities, or nearly 2 million people [21]. The Jewish population itself is multifaceted, representing various ethnicities and identities, from Ashkenazim and Sephardim to Jews of Mizrahi, Ethiopian, and Russian descent [22]. The Arab population represents approximately 20% of the Israeli populace, translating to roughly 1.8 million individuals [23]. The Arab population, ethnically native to the Middle East, has historically supported and practiced consanguineous marriage.

Its different ethnic groups and high rates of endogamy make Israel unique from a genetic point of view. The limited genetic pool, combined with cultural and historical factors that led to marriages within the community and genetic drift, resulted in a high concentration of certain genetic variants. Because of this founder effect, targeted testing for variants previously known to be prevalent in the Israeli population was considered effective. For example, Israel has a population-specific screening program for reproductive purposes that includes all known severe diseases and their relative frequencies in specific subpopulations [24].

Cancer is not a rare disease. According to the Israel Center for Disease Control, in 2020, more than 28,000 individuals were diagnosed with different types of cancer. In the same year, around 12,000 patients died of cancer. As a preventive measure, the Israel Ministry of Health conducted targeted genetic testing for known founder variants in the community to decrease morbidity and mortality.

The present study was conducted in a multiethnic Israeli population, mostly of Ashkenazi Jewish ancestry, attending one of the largest genetic institutes in the country. We sought to determine the yield and implications of targeted genetic testing for cancer in patients and their families. The results showed a notably low, 2%, rate of detection of MHPVs in patients with cancer. Furthermore, only 4% of the healthy individuals with a significant family history of cancer were found to carry MHPVs. The diagnostic yield of MHPVs was slightly higher in the Ashkenazi Jewish group, but still below 5% in all ethnicities. The yield was not more than 10% for all cancer types and did not reach statistical significance. The yield of MHPVs among Ashkenazi Jews with ovarian cancer in this study was only 7%, despite estimates of a carrier risk of ~40% in previous research [25,26], in part, this may be explained by the difference in patients’ characteristics, primarily older age in our cohort, under-representation of patients who had tumor testing first and did not proceed to germline testing, and exclusion of patients who had cascade testing from the cohort. It is worth noting, however, that a larger cohort testing three founder variants in AJ with a significant family history of breast and ovarian cancer showed a similar low yield [9].

We also showed that the inclusion of LPVs in FVT can be problematic. The *APC*:c.3902T>A variant was highly detected among Ashkenazi Jews and frequently detected among non-Ashkenazi Jews; however, there was mostly no clinical correlation or matching to the cancer type or cancer syndrome diagnosed. The variant was tested in individuals of all ethnicities, but it was significant only in Ashkenazi Jews. Based on the recent position statement published by the InSiGHT group, we suggest that the APC:c.3902T>A variant should not be tested in communities other than Ashkenazi Jews [27]. 

Since the LPVs did not explain the clinical presentation in most cases, their detection did not preclude further testing. None of the carriers of this variant in our cohort were reported to have colorectal cancer. However, 22% of the carriers reported a family history of colorectal cancer, similar to findings in previous publications [28,29]. Further research is needed to evaluate the clinical implications of LPVs, particularly in unaffected carriers, in terms of appropriate counseling and risk management with data-driven plans for surveillance and/or risk reduction.

The low yield of targeted testing raises concerns about its efficacy and practicality in a clinical context. Similar findings have been documented in other studies, which emphasize the potential advantages of expanded genetic testing (eGT) over traditional targeted approaches. For instance, the study of Ceyhan-Birsoy et al. [30] showed that the diagnostic yield of eGT varies for different cancer types, but it generally significantly improves the rate of detection of hereditary cancer predisposition compared to targeted testing approaches. It is worth mentioning some obstacles associated with applying eGT to decipher the genetic basis of an inherited predisposition to cancer. Variants of uncertain significance (VUSs) or variants that confer low cancer risk (low-penetrance alleles) may limit the clinical utility and the clinical validity of some variants. In addition, not all pathogenic or likely pathogenic (P/LP) variants are clinically actionable, as there is no evidence for their usefulness in cancer risk reduction.

In our study, the diagnostic yield was significantly higher in patients younger than 40 years. The numbers dropped steeply after 50 years of age, in line with previous publications [31]. It has been established that cancers due to genetic disorders tend to appear earlier in life [31].

Of note are the very low carrier rates detected in non-Ashkenazi Jews and Israeli Arabs. This finding may be partly explained by the different genotypes of these two populations and the lack of optimal representation in the testing panels. For example, in a previous study, the yield of *BRCA1* and *BRCA2* gene sequencing in ethnic Lebanese Arab women with high hereditary-risk breast cancer was much lower than predicted when compared to women of other ethnicities of the same age [32]. Abu-Helalah et al. [32] found that previously reported variants common in Ashkenazi Jews were not prevalent in Jordanian Arab women with breast and ovarian cancer, while other new variants were identified [33]. Further research into the genetic landscape of these populations is needed to optimize the testing panel.

An additional downside of FVT is its limited ability to detect germline PVs in two or more cancer susceptibility genes, known as multilocus inherited neoplasia allele syndrome (MINAS). Testing on individuals carrying these PVs could lead to a false and premature clinical closure, with some PVs going undetected [34]. 

It is noteworthy, based on our experience, that even though targeted genetic testing tends to be less expensive than comprehensive testing, the two-stage testing process often results in a substantial uptake in staff workload. This is due to the increased number of genetic consultations required to communicate the initial results before proceeding to the broader phase of testing. 

## 5. Conclusions

Based on current data, we conclude that FVT in its present form is ineffective. The diagnostic yield is low, even for cancers for which the yield is known to be high in specific populations, such as ovarian cancer in Ashkenazi Jews. We question the advantage of the two-step testing policy because the inclusion of LPVs in the targeted testing, even if positive, does not rule out the need for further comprehensive eGT. If this kind of testing is to be performed, we recommend not including LPVs, and the variants included to be directed to the patient’s clinical characteristics and tumor type.

Given these insights, the necessity for a shift from targeted testing to more comprehensive eGT in clinical practice becomes evident. While some might argue that targeted tests have advantages of specificity and lack of VUS, it is paramount that overall effectiveness be considered in the diagnosis of potential hereditary predispositions. Further research is crucial, but the present study supports a broader approach to genetic testing for cancer predisposition, especially in countries with populations of different ethnicities. Further study of the yield of the second step across this cohort is being planned (suggested bioinformatics pipeline in the Supplementary File S1).

## Figures and Tables

**Table 1:** Variants prevalent in the Israeli population.

Gene	Variant (DNA)	Variant (Protein)	Ethnicity (Frequency)	Gene	Variant (DNA)	Variant (Protein)	Ethnicity (Frequency)
*APC*	c.3920T>A	p.(Ile1307Lys)	AJ (6.5%)	*BRCA2*	c.1813_1814insA	p.(Ile605?fs)	AJ
*BRCA1*	c.2934T>G	p.(Tyr978Ter)	Iranian Jews (1%)	*BRCA2*	c.771_775delTCAAA	p.(Asn257fs)	Muslim Arabs
*BRCA1*	c.181T>G	p.(Cys61Gly)	Russian	*BRCA2*	c.4284dupT	p.(Gln1429Serfs*9)	Muslim Arabs
*BRCA1*	c.224_227delAAAG	p.(Glu75Valfs)	Jewish	*BRCA2*	c.6685G>T	p.(Glu2229*)	Muslim Arabs
*BRCA1*	c.5123C>A	p.(Ala1708Glu)	Jewish	*BRCA2*	c.2482delGACT	Stop770 (exon 11)	Muslim Arabs
*BRCA1*	c.5382insC/c.5266dupC	p.(Gln1756Profs)	AJ (0.45%)	*CHEK2*	c.1100delC	p.(Thr367Metfs)	AJ
*BRCA1*	c.68_69delAG (185delG)	p.(Glu23Valfs)	AJ (0.75%)	*CHEK2*	c.1283C>T	p.(Ser428Phe)	AJ (2.2%)
*BRCA1*	c.981_982delAT	p.(Cys328Terfs)	Jewish	*CHEK2*	c.499G>A	p.(Gly167Arg)	Christian Arabs
*BRCA1*	c.4065_4068delTCAA	p.(Asn1355_Gln1356fs)	AJ	*MLH1*	c.1411_1414delAAGA	p.(Lys471AspfsX19)	AJ
*BRCA1*	c.4153delA	p.(Glu1346fs)	Russian	*MLH1*	c.1771-1772delGA	p.(Asp591Ter)	Jewish
*BRCA1*	c.2311_2317delTTGGTAC	p.(Pro733fs)	Jewish	*MSH2*	c.1906G>C	p.(Ala636Pro)	AJ (0.2%)
*BRCA1*	c.5434 C>G	p.(Pro1812Ala)	Jewish	*MSH2*	c.970_971delCA	p.(Gln324ValfsX8)	Jewish
*BRCA1*	c.3607C>T	p.(Arg1203*)	AJ	*MSH2*	c.1277-1 G>C	IVS7-1G->A	Jewish
*BRCA1*	c.5074+3A>G	IVS17+3A > G	Muslim Arabs	*MSH2*	c.1165C>T	p.(Arg389X)	Jewish
*BRCA1*	c.5444G > A	p.(Trp1815*)	Jewish	*MSH2*	c.705delA	p.(Asp236Thrfs)	Druze
*BRCA1*	c.1224delA	p.(Val409*)	Muslim Arabs	*MSH6*	c.3959_3962delCAAG	p.(Ala1320Glufs)	AJ (0.06%)
*BRCA2*	c.7579delG	p.(Val2527*)	Ethiopian Jews (1%)	*MSH6*	c.3984_3987dupGTCA	p.(Leu330ValfsX12)	AJ
*BRCA2*	c.5946delT/c.6174delT	p.(Ser1982Argfs)	AJ	*MSH6*	c.3603_3606delAGTC	p.(Arg870Serfs*)	Bedouin Arabs
*BRCA2*	c.67+1G>A	IVS2 +1G>A	Jewish	*MUTYH*	c.1187G>A	p.(Gly396Asp)	AJ/Jewish
*BRCA2*	c.7007G>C	p.(Arg2336His)	AJ/Jewish	*MUTYH*	c.536A>G	p.(Tyr179Cys)	AJ/Jewish
*BRCA2*	c.8537_8538delAG/c.8765delAG	p.(Glu2846Glyfs)	Jewish	*MUTYH*	c.1437_1439delGGA	p.(Glu480del)	AJ/Jewish
*BRCA2*	c.3751insA	p.(Thr1251Asnfs)	AJ/Jewish	*PMS2*	c.1970dupA	p.(Asn657LysfsX6)	AJ/Jewish
*BRCA2*	c.3847_3848del/4075delGT	p.(Val1283Lysfs)	AJ	*PMS2*	c.2192T>G	p.(Leu731Ter)	AJ/Jewish
*BRCA2*	c.4936_4939delGAAA	p.(Glu1646Glnfs)	AJ	*PMS2*	c.686_687delCT	p.(Ser229fs)	Muslim Arabs
*BRCA2*	c.4829_4830delTG/5057delTG	p.(Val1610Glyfs)	AJ/Jewish	*TP53*	c.541C>T	p.(Arg181Cys)	Muslim Arabs
*BRCA2*	c.2808_2811delACAA/3036delACAA	p.(Ala938fs)	Jewish				

**Table 2:** Demographic and tumor-related characteristics of the cohort.

	**All individuals**		**Ashkenazi Jewish**		**Partly Ashkenazi Jewish**		**Non-Ashkenazi Jewish**		**Israeli Arabs**	
	Total	%	Total	%	Total	%	Total	%	Total	%
	1758	100	735	42%	173	10%	726	41%	124	7%
**Gender**										
Male	303	17%	140	19%	33	19%	119	16%	11	9%
Female	1455	83%	595	81%	140	81%	607	84%	113	91%
**Age**										
<40	281	16%	125/735	17%	52/173	30%	80/726	11%	24/124	19%
40–50	317	18%	126/735	17%	48/173	28%	101/726	14%	42/124	34%
>50	1160	66%	484/735	66%	73/173	42%	545/726	75%	58/124	47%
**Diagnosis**										
Any cancer	1519	86%	612/1519	40%	127/1519	8%	684/1519	45%	96/1519	6%
Healthy with Family history	239	14%	123/239	51%	46/239	19%	42/239	18%	28/239	12%
**Type of cancer**										
Breast	904	60%	316/904	35%	72/904	8%	443/904	49%	73/904	8%
Prostatic	100	7%	49/100	49%	7/100	7%	41/100	41%	3/100	3%
Colorectal	88	6%	41/88	47%	7/88	8%	35/88	40%	5/88	6%
Ovarian	72	5%	27/72	38%	7/72	10%	34/72	47%	4/72	6%
Pancreatic	64	4%	21/64	33%	7/64	11%	31/64	48%	5/64	8%
Other	291	19%	158/291	54%	27/291	9%	100/291	34%	6/291	2%

**Table 3:** Variant detection rate among cancer patients.

**3a. Variant detection in patients with cancer—All carriers**										
	**TOTAL**		**AJ**		**PARTLY AJ**		**NON-AJ**		**ARABS**	
	**No**	**%**	**No**	**%**	**No**	**%**	**No**	**%**	**No**	**%**
Total	128/1758	7%	73/735	10%	12/173	7%	40/726	5%	3/124	3%
Any Cancer	111/1519	7%	63/612	10%	10/127	8%	35/684	5%	3/96	4%
Healthy & Family History	17/239	7%	10/123	1%	2/46	4%	5/42	12%	0/28	0%
Breast	67/904	7%	36/316	11%	6/72	8%	22/443	5%	4/73	5%
Prostatic	9/100	9%	6/49	12%	1/7	14%	2/41	5%	0/3	0%
Colorectal	6/88	7%	2/41	5%	0/7	0%	4/35	11%	0/5	0%
Ovarian	7/72	10%	3/27	11%	0/7	0%	4/34	12%	0/4	0%
Pancreatic	6/64	9%	2/21	10%	1/7	14%	3/31	10%	0/5	0%
Any Two Cancers	10/76	13%	7/38	18%	1/10	10%	2/19	11%	0/9	0%
Breast and Ovarian	1/6	17%	1/4	25%	0	0%	0/2	0%	0	0%
Breast and Pancreatic	2/5	40%	2/5	40%	0	0%	0	0%	0	0%
Breast and Endometrial	1/11	9%	1/5	20%	0/3	0%	0/3	0%	0	0%
Breast and CRC	1/11	9%	0/7	0%	0	0%	1/4	25%	0	0%
CRC and Endometrial	1/3	33%	1/2	50%	0/1	0%	0	0%	0	0%
CRC and Prostatic	0/7	0%	0/1	0%	0/1	0%	0/2	0%	0	0%
Age < 40	64/281	23%	42/125	34%	8/52	25%	13/80	10%	1/24	4%
Age 40–50	43/317	14%	25/126	20%	2/48	4%	15/101	15%	1/42	2%
Age > 50	21/1160	2%	6/484	1%	2/73	3%	12/545	2%	1/58	2%
**3b. Variant detection in patients with cancer—Carriers excluding low/unknown penetrance variants (APC c.3920T>A, CHEK2 c.1283C>T, and MUTYH heterozygotes).**										
	**TOTAL**		**AJ**		**PARTLY AJ**		**NON-AJ**		**ARABS**	
	**No**	**%**	**No**	**%**	**No**	**%**	**No**	**%**	**No**	**%**
Total	44/1758	3%	29/735	4%	6/173	3%	7/726	1%	2/124	2%
Any Cancer	34/1519	2%	21/612	3%	4/127	3%	7/684	1%	2/96	2%
Healthy & Family history	10/239	4%	8/123	7%	2/46	3%	0/42	0%	0/28	0%
Breast	22/904	2%	13/316	4%	2/72	3%	5/443	1%	2/73	3%
Prostatic	4/100	4%	2/49	4%	1/7	2%	1/41	2%	0/3	0%
Colorectal	0/88	0%	0/41	0%	0/7	0%	0/35	0%	0/5	0%
Ovarian	3/72	4%	2/27	7%	0/7	0%	1/34	3%	0/4	0%
Pancreatic	3/64	5%	2/21	10%	0/7	0%	1/31	3%	0/5	0%
Any two cancers	1/76	1%	1/38	3%	0/10	0%	0/19	0%	0/9	0%
Breast and ovarian	0/6	0%	0/4	0%	0	0%	0/2	0%	0	0%
Breast and pancreatic	0/5	0%	0/5	0%	0	0%	0	0%	0	0%
Breast and endometrial	0/11	0%	0/5	0%	0/3	0%	0/3	0%	0	0%
Breast and CRC	0/11	0%	0/7	0%	0	0%	0/4	0%	0	0%
CRC and endometrial	0/3	0%	0/2	0%	0/1	0%	0	0%	0	0%
CRC and prostatic	0/7	0%	0/1	0%	0/1	0%	0/2	0%	0	0%
Age < 40	20/281	7%	12/125	10%	4/52	8%	3/80	4%	1/24	4%
Age 40–50	17/317	5%	13/126	10%	1/48	2%	3/101	3%	0/42	0%
Age > 50	7/1160	1%	4/484	1%	1/73	1%	1/545	0%	1/58	2%

## Data Availability

The data presented in this study are available on request from the corresponding author. The data are not publicly available due to privacy and ethical issues.

## Supplementary Materials

The following supporting information can be downloaded at: https://www.mdpi.com/article/10.3390/cancers16010094/s1, File S1: Bioinformatics pipeline to be used in the 2nd step of NGS bases expanded genetic testing.

## Abbreviations

eGT	expanded genetic testing
FVT	founder variant testing
LPV	low-penetrance variant
MHPV	medium-high penetrance
MINAS	multilocus inherited neoplasia allele syndrome
NGS	next-generation sequencing
PV	pathogenic variant
