# Supplementary material for: The Diagnostic Yield and Implications of Targeted Founder Pathogenic Variant Testing in an Israeli Cohort
Source: Cancers (Basel). 2023 Dec 24;16(1):94. doi: 10.3390/cancers16010094 (PMC10777957; doi:10.3390/cancers16010094)
Supplement: Supplementary file 1 [file cancers-16-00094-s001.zip › cancers-2721415-supplementary.pdf]

Supplementary File S1

Bioinformatics pipeline to be used in the 2nd step of NGS bases expanded genetic testing

we use FASTQ files that are along with phenotypic information are uploaded into Emedgene's platform (Emedgene Technologies, Mazor, Israel) and analyzed by a team of clinical geneticists and bioinformaticians at our institute, Filtration parameters include variant quality (mapping quality ≥45 and depth ≥10), population frequency (1% or 5% for dominant or recessive inheritance, respectively) and variant impact on the protein.

The list of candidate well-proven cancer predisposing genes focuses on missense, nonsense, frameshift, and splicing variants with a minor allele frequency of <1% in multiple populations. variants will be classified according to the standards and guidelines for sequence variant interpretation of the American College of Medical Genetics and Genomics (ACMG). Variant classification categories include pathogenic, likely pathogenic, variant of uncertain significance (VUS), likely benign, and benign.

P/LP variants are to be classified as having high penetrance (relative risk > 4), moderate penetrance (relative risk 2–4), or low penetrance (relative risk ≤ 2) as well as being recessive or of uncertain clinical actionability.
